# Native language and its connection with mental well-being, emotional state and life satisfaction in a multilingual society

**DOI:** 10.1192/j.eurpsy.2021.1248

**Published:** 2021-08-13

**Authors:** Y. Zinchenko, L. Shaigerova, A. Dolgikh, O. Almazova, R. Shilko

**Affiliations:** Faculty Of Psychology, Lomonosov Moscow State University, Moscow, Russian Federation

**Keywords:** mental well-being, positive and negative affect, multilingualism, native language

## Abstract

**Introduction:**

Ethnolinguistic diversity provides the opportunity to study the relation between the native language, the emotional state, and the well-being of a person. Representatives of different linguistic groups may have psychological advantages in specific socio-cultural situations.

**Objectives:**

We investigated the interrelation between mental well-being, emotional state, life satisfaction, and belonging to different ethnolinguistic categories in the Russian society.

**Methods:**

The measuring instruments included the Warwick-Edinburgh Mental Well-Being Scale (Tennant et al., 2007), the Scale ofPositive and Negative Experience (SPANE) (Diener et al., 2009), the questionary on life satisfaction. The research project included 894 respondents aged from 14 to 80 (M=24.0; SD=11.7), residents of eight regions, where there are one or several official languages along with Russian.

**Results:**

The results indicate that mental well-being (F = 1.167; p = 0.312) is independent of the respondents’ native language, while the ratio of positive and negative affect is significantly higher (F = 3.164; p = 0.008) among people who indicated the regional language as the native one, compared to those who have two native languages - Russian and regional (MD = -1.529; p = 0.039). Moreover, the general life satisfaction is higher (F = 7.427; p = 0.001) among native speakers of the regional language as compared to those who indicate both Russian and regional languages as their native languages (MD = 0.638; p <0.001).

**Conclusions:**

Differences in the emotional state and life satisfaction along with the absence of differences in mental well-being were revealed in respondents of different ethnolinguistic categories. The reported study was funded by RFBR, project number 17-29-09167.

